# Effects of Flow-Induced Shear Stress on Limbal Epithelial Stem Cell Growth and Enrichment

**DOI:** 10.1371/journal.pone.0093023

**Published:** 2014-03-21

**Authors:** Yun Gyeong Kang, Ji Won Shin, So Hee Park, Min-Jae Oh, Hyo Soon Park, Jung-Woog Shin, Su-Hyang Kim

**Affiliations:** 1 Department of Biomedical Engineering, Inje University, Gyeongnam, Korea; 2 Cardiovascular and Metabolic Disease Center, Inje University, Busan, Korea; 3 Nunevit Eye Clinic, Busan, Korea; 4 Department of Health Science and Technology/Institute of Aged Life Redesign/UHRC, Inje University, Gyeongnam, Korea; University of Newcastle upon Tyne, United Kingdom

## Abstract

The roles of limbal epithelial stem cells (LESCs) are widely recognized, but for these cells to be utilized in basic research and potential clinical applications, researchers must be able to efficiently isolate them and subsequently maintain their stemness *in vitro*. We aimed to develop a biomimetic environment for LESCs involving cells from their *in vivo* niche and the principle of flow-induced shear stress, and to subsequently demonstrate the potential of this novel paradigm. LESCs, together with neighboring cells, were isolated from the minced limbal tissues of rabbits. At days 8 and 9 of culture, the cells were exposed to a steady flow or intermittent flow for 2 h per day in a custom-designed bioreactor. The responses of LESCs and epithelial cells were assessed at days 12 and 14. LESCs and epithelial cells responded to both types of flow. Proliferation of LESCs, as assessed using a BrdU assay, was increased to a greater extent under steady flow conditions. Holoclones were found under intermittent flow, indicating that differentiation into transient amplifying cells had occurred. Immunofluorescent staining of Bmi-1 suggested that steady flow has a positive effect on the maintenance of stemness. This finding was confirmed by real-time PCR. Notch-1 and p63 were more sensitive to intermittent flow, but this effect was transient. K3 and K12 expression, indicative of differentiation of LESCs into epithelial cells, was induced by flow and lasted longer under intermittent flow conditions. In summary, culture of LESCs in a bioreactor under a steady flow paradigm, rather than one of intermittent flow, is beneficial for both increasing proliferation and maintaining stemness. Conversely, intermittent flow appears to induce differentiation of LESCs. This novel experimental method introduces micro-mechanical stimuli to traditional culture techniques, and has potential for regulating the proliferation and differentiation of LESCs *in vitro*, thereby facilitating research in this field.

## Introduction

The importance and potential of limbal epithelial stem cells (LESCs) in ophthalmology are widely recognized. Transplanted LESCs, like most types of stem cells, are useful for the treatment of partially or completely damaged tissues. They can influence processes such as conjunctivalization, vascularization, and chronic inflammation [1]–[4]. LESCs play an important role in the long-term maintenance of epithelial cells in the central region of the cornea [5]. Corneal epithelial cells may be replaced by new epithelial cells within 1–2 weeks [6]. To replace epithelial cells *in vivo*, LESCs are believed to continuously proliferate and differentiate into transient amplifying cells (TACs) [7]. Another study found that the proliferative capacity of epithelial cells was diminished when the limbal epithelium was eliminated [8].

For LESCs to be utilized in basic research and potential clinical applications, researchers must be able to efficiently isolate them and subsequently maintain their stemness and other phenotypic characteristics *in vitro*. Several techniques of isolating LESCs for subsequent primary culture have been reported, including the “side population” method and the general isolation technique [9], [10]. The side population approach enables the acquisition of LESCs with relatively high purity, but this protocol involves many complicated steps, and obtaining sufficient cells can be challenging. The general isolation technique usually involves the dissection of stromal tissue and the endothelial sheath, followed by collection of LESCs from the epithelial region. Unfortunately, this method also yields an insufficient quantity of LESCs. Furthermore, pure cultures of isolated LESCs proliferate more slowly *in vitro* [11]. Recently, another procedure was reported, in which LESCs were isolated and cultured together with cells from their *in vivo* niche [12]. More observable holoclones and meroclones were formed under these conditions, and the expression of stem cell markers, including Vimentin, Oct4, Sox2, Nanog, Rex1, Nestin, N-cadherin, SSEA4, and CD34, was upregulated. Hence, native niche cells are believed to play a role in maintaining the characteristics of LESCs *in vitro*.

We adopted a similar procedure for acquiring LESCs together with neighboring cells. Minced limbal tissues were digested in a solution of dispase II. Dissociated cells were then obtained by centrifugation and cultured for up to 14 days. The premise of our culture protocol was that cells from limbal niches may provide a permissive environment in which LESCs can proliferate and maintain their *in vivo* phenotype, even after they are isolated from tissues. We also introduced flow-induced shear stress to our culture paradigm. Mechanical factors are recognized to influence the ability of LESCs to maintain their functions and characteristics [13], [14]. Understanding the roles of blinking and intraocular pressure is essential in studying LESC micro-mechanical environments [6]. Mather and Daley reported that on average, the blinking rate in humans is 20 blinks/min and the tear volume turnover rate is 0.31 μL/min [15]. Tear flow due to blinking induces shear stress on the surface of the eye. Therefore, we designed and utilized a bioreactor system with the capacity to apply flow-induced shear stress to cells to investigate the effects of shear stress on LESCs and/or immature cells.

In summary, we isolated LESCs together with neighboring cells from their *in vivo* niche and cultured them in a system in which they were exposed to flow-induced shear stress. The effects of flow-induced shear stress on the proliferation and maintenance of stemness in LESCs were then investigated using various biological assays.

## Materials and Methods

### Isolation and Culture of Limbal Epithelial Stem Cells

New Zealand white rabbits (8 weeks old, 1.5–2.0 kg) were purchased from Samtako (Kyoung-Ki, Korea). All experimental procedures were based on the ARVO Statement for the Use of Animals in Ophthalmic and Vision Research and were approved by the Institutional Animal Care and Use Committee of Inje University. Extracted eyeballs were washed three times in phosphate-buffered saline (PBS) containing 1% penicillin–streptomycin. The limbal area was cut into a ring by removing the cornea, iris, sclera, and other miscellaneous tissues. The minced limbal tissues were immersed in dispase II solution for 1.5 h and then treated with trypsin–EDTA. NIH3T3 fibroblasts (feeder cells), treated with mitomycin C (4 μg/mL; Sigma, St. Louis, MO) for 2 h, were seeded into 6-well plates (2×10^4^ cells/cm^2^). Cells obtained from dissociated limbal tissues were seeded onto the feeder cells (1×10^4^ cells/cm^2^) and then cultured in an incubator at 37°C in an atmosphere containing 5% CO_2_ and 95% air. Cultures were maintained in supplemented hormonal epithelial medium (SHEM): Dulbecco's modified Eagle's medium (DMEM)/F12 (1∶1; Life Technologies, Carlsbad, CA) containing 5% fetal bovine serum (FBS), 0.5% dimethyl sulfoxide (DMSO; Sigma), 10 ng/mL human epidermal growth factor (hEGF; Sigma), ITS Premix (BD Biosciences, Lincoln Park, NJ), 0.5 μg/mL hydrocortisone (Sigma), 50 μg/mL gentamicin (Sigma), and 1.25 μg/mL amphotericin B (Sigma).

### Bioreactor system for Inducing Shear Stress

**Figure 1:**
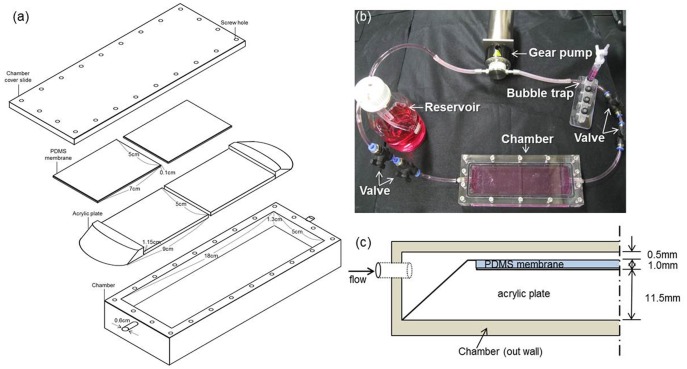
Bioreactor for flow engaging (a) Parts of the flow chamber used in this study. (b) Components of the system. The bubble trap was used to eliminate any bubbles. (c) Side-view of the assembled chamber (not to scale).

We designed a bioreactor system (Figure 1) that enables the cells to experience flow-induced shear stress intended to mimic the effect of blinking on the eyeball. A minor modification was made to the previously described system [16]. The system utilizes a general incubator and consists of a gear pump (REGKI-ZS Digital, ISMATEC; IDEX Corporation, Lake Forest, IL), a reservoir, a bubble trap, and a custom-designed chamber. The inner dimensions of the chamber are as follows: 18.0 cm length, 5.0 cm width, and 1.3 cm height. Two acrylic plates (7.00×5.00×1.15 cm^3^) could be placed inside the chamber (Figure 1A).

### Seeding and Flow Engaging

Using a spin-coating technique, each acrylic plate was coated with a 1.0-mm layer of polydimethylsiloxane (PDMS; Sylgard 184 Silicone Elastomer Kit; Dow Corning, Midland, MI). The PDMS surfaces were treated with plasma (APP Co., Ltd., Suwon, Korea) and then coated with fibronectin (1∶100; Sigma), before seeding with feeder cells (NIH3T3 cells, 2×10^4^ cells/cm^2^) that had been pretreated with mitomycin C (2.5 μg/mL). Cells from limbal tissues were then seeded uniformly onto the feeder cells on each plate. Four hours later, the PDMS plates were transferred to a square dish (SPL Life Sciences, Pochon, Korea) containing SHEM medium (80 mL/dish) and cultured in an incubator.

**Figure 2:**
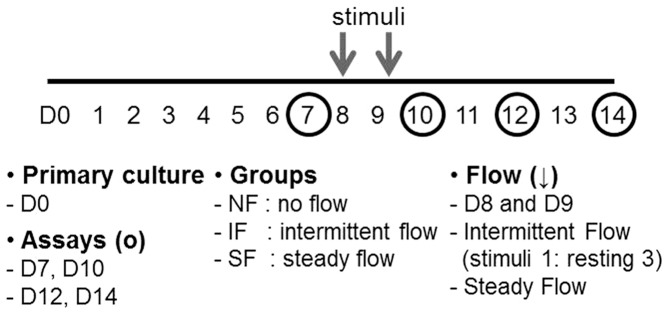
Experimental schedule and nomenclature for corresponding experimental groups.

In preliminary experiments, down-regulation of LESC and epithelial marker gene expression, as assessed by real-time PCR, was observed at day 10, compared to day 5 (data not shown). Therefore, we applied shear stress at day 8, before gene expression decreased. Briefly, at day 8 of culture, two acrylic plates were inserted into the chamber of the bioreactor system and flow-induced shear stress was applied. The flow rate was set to 0.93 mL/min. Two types of flow were used: steady and intermittent. Both were applied for 2 h/day for 2 days (day 8 and 9). Intermittent flow consisted of a 1 min on, 3 min off cycle [16]. Three experimental conditions were studied: no flow (NF), steady flow (SF), and intermittent flow (IF). The full experimental paradigm is illustrated in Figure 2.

### BrdU Labeling

Bromodeoxyuridine (5-bromo-2′-deoxyuridine; BrdU) is known to be incorporated into the newly synthesized DNA of replicating cells during the S phase of the cell cycle. To identify actively replicating cells, and thereby assess cellular proliferation, BrdU was applied to cells at days 7, 10, and 12 of culture. To visualize BrDU, cultures were incubated for 2 h with a monoclonal anti-BrdU antibody (clone BU 33; Sigma). After fixation with methanol for 10 min, the cells were treated with 1 N HCl for 1 h at room temperature before incubation with a TRITC-conjugated secondary antibody (Sigma) for 1 h in a dark room. Finally, the cells were stained with 1 mg/mL 4′,6-diamidino-2-phenylindole (DAPI; Vectashield; Vector Laboratories, Burlingame, CA). Immunofluorescence was observed using a confocal microscope (Carl Zeiss, Jena, Germany) and images were acquired using AxioVision software (Carl Zeiss). Images (n = 3 for each experimental conditions) were converted to binary and analyzed using ImageJ software (version 1.44; National Institutes of Health, Bethesda, MD).

### Visualization of Colonies Using Rhodamine B Staining

To visualize cell colonies, cultures at day 14 were fixed with 4% paraformaldehyde for 30 min at room temperature and then incubated with 1% rhodamine B (Sigma) for 30 min. Staining was observed using a confocal microscope (Zeiss Axiovert 200 M; Carl Zeiss) with a CCD camera.

### Real-time PCR

Real-time PCR was conducted using a 7500 Real-Time PCR System (Applied Biosystems, Foster City, CA) to investigate the presence of specific markers at days 7, 10, and 14. Primers are described in Table 1. The expression of each marker was normalized to that of GAPDH and data are presented as the mean and standard deviation (SD). Data analysis was performed using 7500 System Sequence Detection software (Applied Biosystems) according to the 2^−ΔΔCt^ method. PicoGreen DNA quantification and real-time PCR analyses were performed three times for each sample.

**Table 1:** Primers used in quantitative real-time RT-PCR.

Cell type	Gene Product	Forward primer (5′→3′)	Reverse primer (5′→3′)	Accession No.
**Limbal epithelial stem cells**	Notch-1	CCGTGACCGCTGAGAACAT	CACGGGTGTGCTGATACTTCTG	XM002720852.1
	p63	GGATTCCCCTGAGTGCTGTCT	TCCCAGGGAAGGCAAACTT	XM002716505.1
	Bmi-1	ACGATGCCCAGCAGCAAT	TTAGAGCCATTGGCAGCATC	AB231854.1
**Epithelial cells**	K3	ACAACCTCGAGCCGCTTTTT	GTCTTCCCCTCCCTCCCTTC	S65740.1
	K12	GGGAGGCCCAAGGTGATG	TTGAGACCTGGAGCCTGTCAT	X77665.1
**All cells**	GAPDH	GTCGTCTCCTGCGACTTCAA	GGAGGCAGGGATGATGTTCT	A231852.1

### Immunofluorescence Microscopy

Cells from each experimental group were immunolabeled with antibodies against Bmi-1, K3, and K12. K3 and K12 were used to identify differentiated epithelial cells. Cells were immersed in PBS containing 1% bovine serum albumin (BSA) for 30 min to suppress nonspecific binding. The secondary antibody used was a TRITC-conjugated goat anti-mouse antibody. Bmi-1, K3, and K12 immunofluorescence was quantified Using ImageJ software by converting images to binary and calculating the area of positive fluorescence (n = 7–10).

### Statistical Analysis

Data are presented as the mean ± SD. Data for different groups were analyzed using a one-way analysis of variance (ANOVA) with *post hoc* testing (Fisher's least significant difference test) using PASW Statistics software (SPSS Inc., Chicago, IL). Differences were considered to be statistically significant at *p*<0.05.

## Results

### Proliferation of LESCs

**Figure 3:**
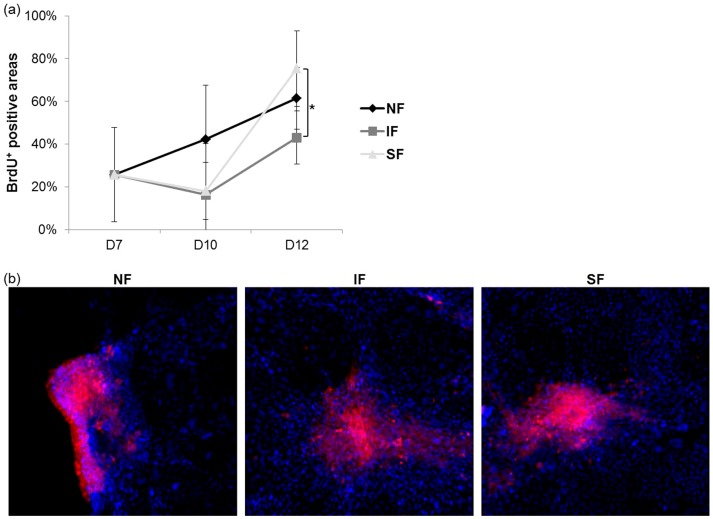
BrdU staining. (a) The percentages of stained areas calculated from digitized images (n = 3; *p<0.05). (b) Representative images at day 12 (100×).

The percentage of the culture area in each experimental group that was positive for BrdU staining is shown in Figure 3, in addition to representative images of cultures stained at day 12. A steady increase in BrdU staining was found in the group with no flow (NF group), suggesting steady proliferation. At day 10, shear stress suppressed proliferative activity, regardless of whether it was caused by interrupted flow (IF group) or steady flow (SF group). Note that the flow was engaged on days 8 and 9. The suppressive effect on proliferation was temporary under both the interrupted flow and steady flow conditions and was comparable to that of the NF group at day 12. The SF group showed higher proliferative activity than the NF group, although no significant difference was found. These results indicate that flow-induced shear stress affects proliferation of LESC cultures.

### Morphology and LESCs Marker Expression

**Figure 4:**
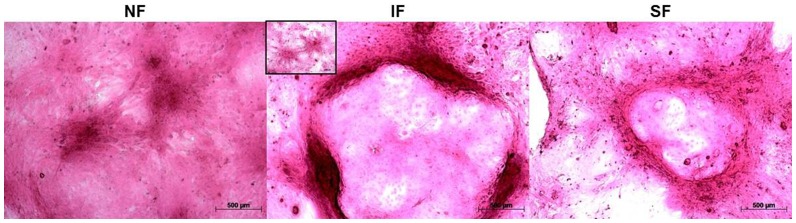
Typical images of rhodamine B staining for each group at day 14 (50×). Holoclone shapes were found in the IF group, indicating differentiation into TACs. Inset panel: typical meroclone morphology under intermittent flow (50×).

**Figure 5:**
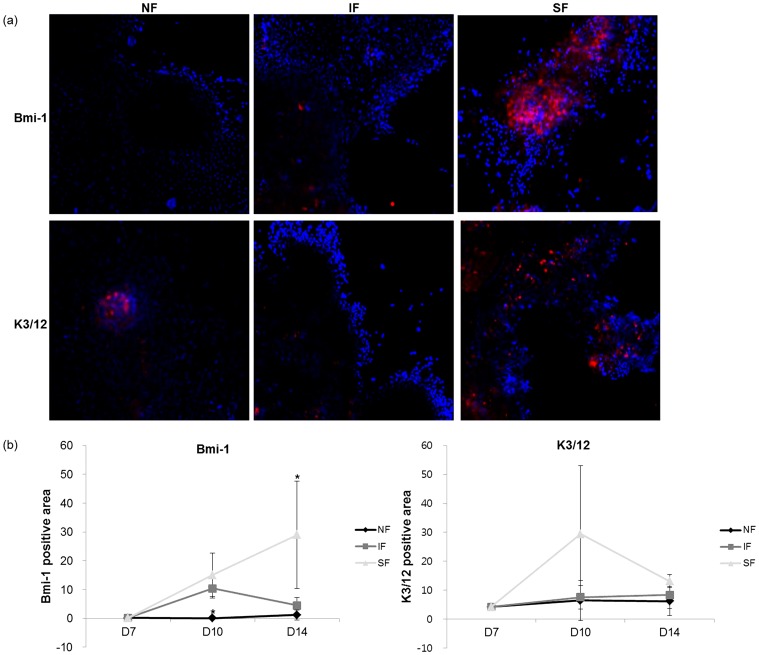
Immunofluorescence staining. (a) Representative images of immunofluorescence staining at day 14 (100×). (b) Percentage of stained area, calculated from digitized images (n = 7–10, *p<0.05).

Colonies stained with rhodamine B at day 14 are shown in Figure 4. Colonies in the IF and SF groups were significantly larger than those in the NF group. Notably, holoclone shapes were found in the IF group, indicative of differentiation into TACs. The results of immunofluorescence staining for Bmi-1, K3, and K12 are shown in Figure 5. The NF group showed no difference in the expression of these markers throughout the experimental time-course (Figure 5b). Bmi-1 expression was more strongly affected by flow. Steady flow increased expression of Bmi-1 continuously, even after flow stopped. The effect of intermittent flow on the expression of Bmi-1, however, was temporary. Expression of the epithelial cell markers (K3 and K12) were strongly affected immediately after steady flow shear stress, but the effect decreased over time (Figure 5b). These results suggest that steady flow conditions are beneficial in maintaining the stemness of LESCs *in vitro*, without having a lasting effect on the expression of K3 and K12.

### Gene Expression of LESCs and Epithelial Cell Markers

**Figure 6:**
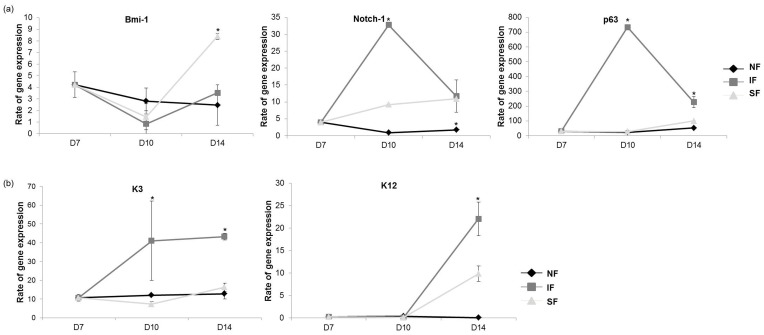
The effects of shear stress on mRNA expression of various cell markers. Target mRNA expression was normalized relative to the GAPDH signal. (a) LESC-related markers (Bmi-1, Notch-1, and p63). (b) Epithelial cell-related markers (K3 and K12) (n = 3, *p<0.05).

The gene expression of various markers related to LESCs or epithelial cells are shown in Figure 6. Little change was observed in all markers studied when the cells were cultured without flow. Exposure to flow affected gene expression, and in general, intermittent flow had a greater effect than steady flow. Among LESC-related markers, the expression of Notch-1 was dramatically increased immediately after intermittent flow, although this effect did not last long. The same trend was observed for the expression of p63. The effect of flow on the expression of Bmi-1 was found to be somewhat different from the effect on Notch-1 and p63. Immediately after the flow was stopped, the expression of Bmi-1 tended to decrease, but this was followed by an eventually increase in expression, particularly in the case of the steady flow paradigm. The expression of K3 and K12 was also affected by flow. In the absence of flow, the expression levels were consistent, but expression was markedly affected by intermittent flow.

## Discussion

This study aimed to show the potential of a new methodology for expanding LESCs *in vitro* while maintaining their stemness. The premise is that a favorable *in vitro* environment is likely to be similar to that which the cells experience in their *in vivo* niche. Therefore, we isolated and cultured LESCs together with neighboring cells, which we expected to provide biological conditions similar to those of the *in vivo* environment. Previous studies have shown that culturing LESCs with neighboring cells, rather than culturing them alone, results in more positive outcomes [12]. In addition to the neighboring cells, we added another factor: flow-induced shear stress. Engagement of shear stress was intended to mimic micro-mechanical environment of a blinking eye. Various kinds of mechanical stimuli, such as compression, tension, and flow-induced shear stress, have been widely adopted in the research areas of general stem cells, and their roles have been recognized [17]–[22]. When applying mechanical stimulation to cells, magnitude and frequency are important. Tensile stimulation of mesenchymal stem cells, even without any biochemical reagents and/or growth factors, has been demonstrated to promote differentiation into osteoblasts or smooth muscle cells, depending on the magnitude of force [23]. However, no experimental research concerning mechanical stimulation of LESCs has been reported. This study is the first time that flow-induced shear stress has been adopted as a paradigm for mechanical stimulation for LESCs.

A low value for the Reynold's number enabled us to calculate the magnitude of the shear stress based on fully developed Newtonian laminar flow [24]. The equation used is τ_mean_ = 6 μQ/bh^2^, where μ (9.6×10^−4^ Pa•s) is the dynamic viscosity of the culture medium, Q is the flow rate (in this study 0.93 mL/min, the minimum rate controllable using the pump), and b (50 mm) and h (0.5 mm) are the width and height of the cross section in the chamber, respectively. The magnitude of the shear stress was calculated to be 7.1×10^−3^ Pa. One study found that the *in vivo* tear flow turnover rate is 0.31 μL/min [15]. Based on this previous report, the magnitude of the shear stress can be calculated to be 5.0×10^−3^ Pa when the width of an eyeball and clearance between the eyelid and eyeball are assumed to be 15.0 mm and 20.0×10^−3^ mm, respectively. However, this calculation was based on the tear flow turnover rate alone and did not take into account the speed of blinking and other pressurizing effects drying eyelid movement. Therefore, a 40% deviation from the theoretically calculated magnitude could be acceptable, considering that all other factors were assumed.

In addition to the magnitude of the shear stress, its temporal pattern is also important. For example, the duration of opening and closing may differ from each other. Indeed, Jones *et al*. reported that the closing speed of the eyelid is at most three times faster than the opening speed [25]. Assuming the ratio is three to one, we applied flow in a 1 min on, 3 min off cycle to induce intermittent shear stress.

Even though the effects of mechanical stimuli have been widely recognized in most cell types, the optimal magnitude and frequency of any stimulation on a certain cell type have not yet been clarified. Moreover, no research of this nature has been conducted in the field of ophthalmology. Replicating the pattern of mechanical stimulation that a given cell or tissue experiences *in vivo* may be the best approach; however, whether this is truly necessary once cells have been isolated from tissues and cultured *in vitro* is uncertain. We therefore set out to investigate the effects of flow-induced shear stress on the responses of LESCs *in vitro*.

The results of BrdU staining, an indicator of cell proliferation, showed that proliferation was affected by flow. The steady proliferation evident in the group without flow confirmed that the cells were handled properly. However, we observed a decreased proliferation rate immediately after engagement of flow at day 10. Note that the flow was engaged at day 8 and 9 for 2 h per day. Importantly, exposure to flow-induced shear stress resulted in increased proliferation, particularly in the case of a steady flow paradigm.

Larger colonies were found in the flow-stimulated groups compared to the control group (Figure 4). Meroclones and holoclones were found in the group subjected to intermittent shear stress, indicative of differentiation into TACs (Figure 4). This observation suggests that intermittent flow stimulation may promote LESC differentiation.

Immediately after stimulation was stopped, expression of Notch-1 and p63, a putative stem cell marker and a proliferation marker, respectively, dramatically increased. This upregulation was sustained following intermittent shear stress, but tended to continue to increase following steady flow stimulation. The expression of Bmi-1 increased 3–4 days after stimulation was stopped. These findings provide evidence that steady flow is better than intermittent flow in terms of stimulating proliferation and maintaining LESC characteristics. Meanwhile, the expression of K3 and K12, markers of corneal epithelial cells, was predominantly affected by intermittent shear stress, suggesting that intermittent flow may play a role in cell differentiation. The effects of flow were also confirmed at the protein level: the effect of steady flow on LESC protein expression persisted, and intermittent flow had a marked effect on the expression of K3 and K12.

Our experiments were the first to apply mechanical stimulation in the form of flow-induced shear stress to the culture of LESCs. These flow paradigms, which were designed to mimic physical conditions (the effect of tear flow turnover due to blinking), showed potential for stimulating proliferation and maintaining the stemness of LESC cultures. The effects of mechanical stimulation were dependent on the type of flow. A steady flow paradigm appears to be preferable for the maintenance and expansion of LESCs. Conversely, intermittent flow induces the differentiation of LESCs into epithelial cells. Some limitations exist, however, to the research described here. Consequently, further experiments are needed to develop a concrete protocol for the control of proliferation and differentiation of LESCs by adjusting the magnitude and frequency of shear stress.
